# Non‐Stationary Complementary Non‐Uniform Sampling (NOSCO NUS) for Fast Acquisition of Serial 2D NMR Titration Data

**DOI:** 10.1002/anie.202009479

**Published:** 2020-09-29

**Authors:** Javier A. Romero, Ewa K. Nawrocka, Alexandra Shchukina, Francisco J. Blanco, Tammo Diercks, Krzysztof Kazimierczuk

**Affiliations:** ^1^ Centre of New Technologies University of Warsaw Banacha 2C 02-097 Warsaw Poland; ^2^ Faculty of Chemistry University of Warsaw Pasteura 1 02-093 Warsaw Poland; ^3^ Faculty of Chemistry Biological and Chemical Research Centre University of Warsaw Zwirki i Wigury 101 02-089 Warsaw Poland; ^4^ Structural and Chemical Biology Department Centro de Investigaciones Biológicas CIB-CSIC 28040 Madrid Spain; ^5^ CIC bioGUNE Parque Tecnológico de Bizkaia, Ed. 800 48160- Derio Spain

**Keywords:** compressed sensing, ligand binding, NMR spectroscopy, non-uniform sampling, titration

## Abstract

NMR spectroscopy offers unique benefits for ligand binding studies on isotopically labelled target proteins. These benefits include atomic resolution, direct distinction of binding sites and modes, a lowest detectable affinity limit, and function independent setup. Yet, retracing protein signal assignments from apo to holo states to derive exact dissociation constants and chemical shift perturbation amplitudes (for ligand docking and structure‐based optimization) requires lengthy titration series of 2D heteronuclear correlation spectra at variable ligand concentration that may exceed the protein's lifetime and available spectrometer time. We present a novel method to overcome this critical limitation, based on non‐stationary complementary non‐uniform sampling (NOSCO NUS) combined with a robust particle swarm optimization algorithm. We illustrate its potential in two challenging studies with very distinct protein sizes and binding affinities, showing that NOSCO NUS can reduce measurement times by an order of magnitude to make such highly informative NMR titration studies more broadly feasible.

## Graphical abstract

A novel complementary non‐uniform sampling scheme (NOSCO‐NUS) and processing algorithm (NOSCO) massively speed up the recording of complete series of 2D NMR titration spectra to obtain accurate affinity constants and structural constraints for ligand docking within less than 10 % of the ordinary time.

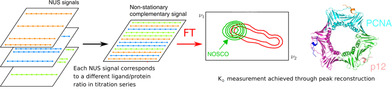

Solution state NMR spectroscopy is a most powerful analytical technique to study molecular interactions with atomic resolution, down to the lowest affinities (*K*_A_≥10^2^ M^−1^), without a function‐specific set‐up nor risk of bias from biochemical side reactions.^[1]^ Thus, NMR can greatly help to elucidate protein function, by identifying even weakly interacting partners, their pertaining binding sites, and the functional groups involved in the molecular interaction, and is now a preferred technique for sound and unbiased studies of multifaceted protein/protein interactions. With its unique combination of strengths, NMR is furthermore employed by pharmaceutical industry to access novel ligand classes, most efficiently by screening a manageable library of chemically diverse molecular fragments^[2]^ that typically bind too weakly for detection by any other technique. By simultaneously reporting on affinity and atomic details of interaction, NMR then helps to elucidate structure–activity relationships^[3]^ and guide the iterative structure‐based optimization^[4]^ into more complex, specific, high‐affinity ligands (structure‐based drug design, SBDD). This powerful NMR application requires large amounts of isotopically labelled (^15^N or ^13^C) target protein and long measurement times to record well‐resolved heteronuclear 2D correlation spectra of the protein, where the signals of amino acids affected by ligand binding gradually shift or disappear with increasing ligand concentration. The time exigency is exacerbated by the need to record full titration series to determine the affinity constant, retrace signal assignments from the *apo* to *holo* state, and quantify the chemical shift perturbation (CSP) amplitude[^5^, ^6^] of individual signals as the most accurate directly accessible NMR constraint for ligand docking (e.g., by HADDOCK^[7]^) and structure‐based ligand optimization. Here we present a new method to massively speed up such exhaustive interaction studies without loss of any information. Complete titration series of 2D protein NMR spectra can, thus, be recorded within less than a day, making them more broadly applicable, e.g., in industrial SBDD processes, or for proteins with limited lifetime.

The critical information on molecular dissociation constants, *K*_D_, and chemical shift perturbation (CSP) amplitudes, Δ*v*, can be derived by individually fitting the correlated signal frequencies, observed in the 2D protein NMR spectra of a titration series, to the equation [Eq. 1]:

(1) $$\nu_{I}=\nu_{I,0}+f\left(x,K_{D}\right)\cdot \Delta \nu_{I}$$

where [Eq. 2]

(2) $$f\left(x,K_{D}\right)=\;\frac{K_{D}+\left(1+x\right)c_{P}-\sqrt{{\left(K_{D}+\left(1+x\right)c_{P}\right)}^{2}-4c_{P}^{2}x}}{2c_{P}}$$

*v*_I_ is the observed chemical shift (I=^15^N or^1^H ), *v*_I,0_ is the pertaining reference chemical shift in the absence of ligand; Δ*v*_I_ is the maximal CSP *amplitude* due to ligand binding; *x*=*c*_L/_ *c*_P_ is the ligand to protein molar ratio; *c*_P_ is the total protein concentration.

NMR frequency sampling in an indirect time dimension is a lengthy process (fundamental reasons described in SI). Here we propose a method for complementary non‐uniform sampling (NUS) of entire titration series of 2D ^1^H‐^15^N HSQC spectra within less time even than required for a *single* conventional spectrum. For this we sample the indirect *t*_1_(^15^N) dimension as in a standard NUS experiment, yet with complementary sampling schedules for the different titration points such that each ligand‐to‐protein ratio, *x*, is sampled for a unique set of *t*_1_ times (Figure 1). A full grid of 256 *t*_1_ increments in a titration series with 8 different *x* ratios would, thus, be sampled in 8 complementary schedules of 32 points. The amount of data to be acquired could be further reduced by combination with compressed sensing (CS) reconstruction.[^8^, ^9^] The various NUS 2D time‐domain data of a titration series can be combined into a 3D matrix and co‐processed to yield a single 2D HSQC spectrum, which represents the projection along the third *pseudo* dimension encoding the ligand‐to‐protein ratio, *x*. While signals unaffected by the added ligand maintain their position and shape (provided that a possible dilution effect is compensated for by appropriate intensity scaling), signals that shift strongly (i.e. show large CSP) appear smeared out and broadened due to the superposition of their variable positions in the different underlying 2D spectra of the titration series. The line shape modulation of such signals with strong CSP was described in detail in our previous work.^[10]^ A well‐separated NMR signal in the co‐processed 2D spectrum of a full titration series can be mathematically expressed (neglecting relaxation) as [Eq. 3]:

(3) $$S_{\mathrm{NS}}\left(x\left(t_{1}\right),t_{1},t_{2}\right)=\;A\left(x\left(t_{1}\right)\right)\cdot \exp\left(i2\pi \nu_{N}\left(x\left(t_{1}\right)\right)t_{1}+i2\pi \nu_{H}\left(x\left(t_{1}\right)\right)t_{2}\right)$$

**Figure 1:**
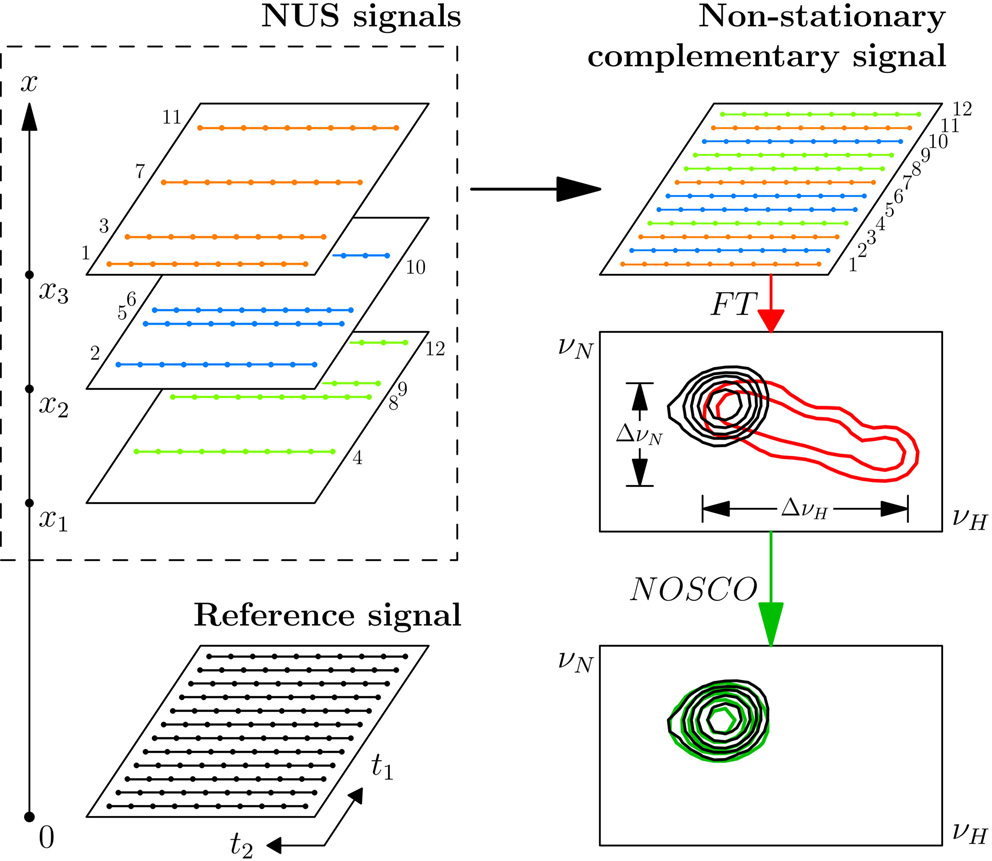
The concept of non‐stationary complementary (NOSCO) sampling of 2D NMR spectra in titration series and signal processing. A reference spectrum is measured separately (with or without NUS) before adding ligand. Titration spectra are then sampled at different ligand‐to‐protein ratios (*x*) and with complementary NUS schedules, where *x* acts as a third pseudo dimension in the resulting pseudo‐3D time domain data matrix. The combination of signals and subsequent Fourier transform produces a single 2D correlation spectrum in which signals appear broadened if they experience strong CSP during the titration series (red contours). NOSCO processing effectively refocuses such non‐stationary signals (green contours) into their corresponding reference signal (black contours).

*S*_NS_ is a *non‐stationary* signal^[10]^ if its correlated frequencies *v*_N_ and *v*_H_ change with the ligand‐to‐protein ratio *x*, which ultimately depends on the indirect sampling time *t*_1_ via the chosen NUS schedule. *A*(*x*(*t*_1_)) represents a possible signal amplitude scaling by dilution. With the proposed non‐stationary *complementary* (NOSCO) NUS schedule proposed above, the *x*(*t*_1_) dependence becomes straightforward, unambiguous, and traceable.

A 2D time‐domain signal *S*_NS_(*t*_1_,*t*_2_) can be obtained by Inverse Fourier Transform (IFT) of a single‐peak spectral region containing only its frequency‐domain counterpart, ${\tilde{S}}_{\mathrm{NS}}\left(\nu_{1},\nu_{2}\right)$ . The single‐site binding model [Eq. (1)] predicts that *S*_NS_ [Eq. (3)] is the product of a stationary component with frequencies *v*_I,0_ and a non‐stationary component depending on *f*(*x*(*t*_1_),*K*_D_) as well as the CSP amplitude Δ*v*_I_. The time‐domain counterpart of each peak in a co‐processed titration series spectrum acquired with complementary NOSCO sampling therefore is the product of a regular (stationary) signal from the reference sample (without ligand) and a non‐stationary factor (dependent on the ligand‐to‐protein ratio) that always causes signal broadening and attenuation.

The idea behind NOSCO processing is to remove the *non‐stationary* factor for each *smeared* peak, thus refocusing it into the sharp reference signal with a concomitant increase in intensity (Figure 1). For this, NOSCO searches a combination of non‐stationarity parameters {Δ*v*_N_,Δ*v*_H_,*K*_D_} that produces a correction signal *S*_C_ for each *smeared* 2D time‐domain signal *S*_NS_ with minimal difference to its reference signal [Eq. 4]:

(4) $$\underset{\Delta v_{N}\Delta v_{H}K_{D}}{\arg\min}\left|\max\left\{\mathrm{FT}\left[S_{\mathrm{NS}}\cdot S_{C}\right]\right\}-\max\left\{\mathrm{FT}\left[S_{P}\right]\right\}\right|$$

FT is the Fourier Transform of the time domain product (*S*_NS_⋅*S*_C_), max is the maximum value of the transform (the peak height), *S*_P_ is the reference signal in an HSQC spectrum acquired separately (with or without NUS) before ligand addition. The correction signal *S*_C_ depends on the adjustable CSP amplitudes (Δ*v*_N_, Δ*v*_H_) and dissociation constant (*K*_D_) as follows [Eq. 5]:

(5) $$S_{C}=\exp\left(-i2\pi \cdot f\left(x\left(t_{1}\right),K_{D}\right)\cdot \left(\Delta \nu_{N}\cdot t_{1}+\Delta \nu_{H}\cdot t_{2}\right)\right)$$

As Equation (4) may have several local minima, its global minimization requires to efficiently scan a vast parameter space {Δ*v*_N_, Δ*v*_H_, *K*_D_} for each selected *smeared* peak. For this purpose, we implemented a Particle Swarm Optimization (PSO) algorithm^[11]^ that can simultaneously optimize the parameter triplet {Δ*v*_N_, Δ*v*H, *K*D} without requiring the target function to be smooth, and with robustness against falling into local minima (see SI for more information).

We have applied the proposed NOSCO NUS method to two protein‐ligand systems with quite distinct protein sizes, binding constants, signal‐to‐noise ratios, and spectral dispersions. The first protein, SH3 domain of α‐spectrin, has 60 residues and binds proline‐rich peptides by recognizing sequences with a PXXP motif.^[12]^ As ligand we used the short p41 decapeptide[^13^, ^14^] and screened *n*_x_=8 ligand‐to‐protein ratios *x*. NOSCO NUS sampling of the full pseudo 3D data, thus, affords a compaction of the overall measurement time by factor *n*_x_=8, given the same overall sampling level as for each individual 2D titration spectrum. At pH 3.5, SH3 binds p41 very weakly (*K*_D_=9 mM) and, therefore, requires high ligand‐to‐protein ratios for reliable *K*_D_ determination. The results from conventional fitting of Equation (1) to the observed CSP values of selected SH3 domain signals are shown in Figure 2. Despite the low protein concentration used, no CSP curve reaches near its plateau value, making *K*_D_ determination challenging by any method. On the other hand, the 2D ^1^H‐^15^N HSQC spectrum of SH3 domain remains rather simple and well‐resolved throughout the titration series, despite some clear signal *smearing* in the NOSCO spectrum. This high spectral sparsity facilitates the *undersampling* mode of NOSCO NUS (see above and SI). The second protein, human Proliferating Cell Nuclear Antigen (PCNA), forms a symmetrical homotrimer of 87 kDa that binds many proteins, including the polymerase‐*δ* p12 subunit. As ligand we used a fragment of p12^[15]^ and *n*_x_=6 ligand‐to‐protein ratios *x*, implying a potential time saving by NOSCO NUS of at least a factor *n*_x_=6. In contrast to the SH3/p41 system, PCNA/p12 binding is stronger (*K*_D_=130 μM), close to the limit for biologically relevant interactions, while spectral intensities and sparsity are much lower for PCNA. Therefore, each 2D titration spectrum required 22 h measurement time (vs. 1.5 h for SH3).

**Figure 2:**
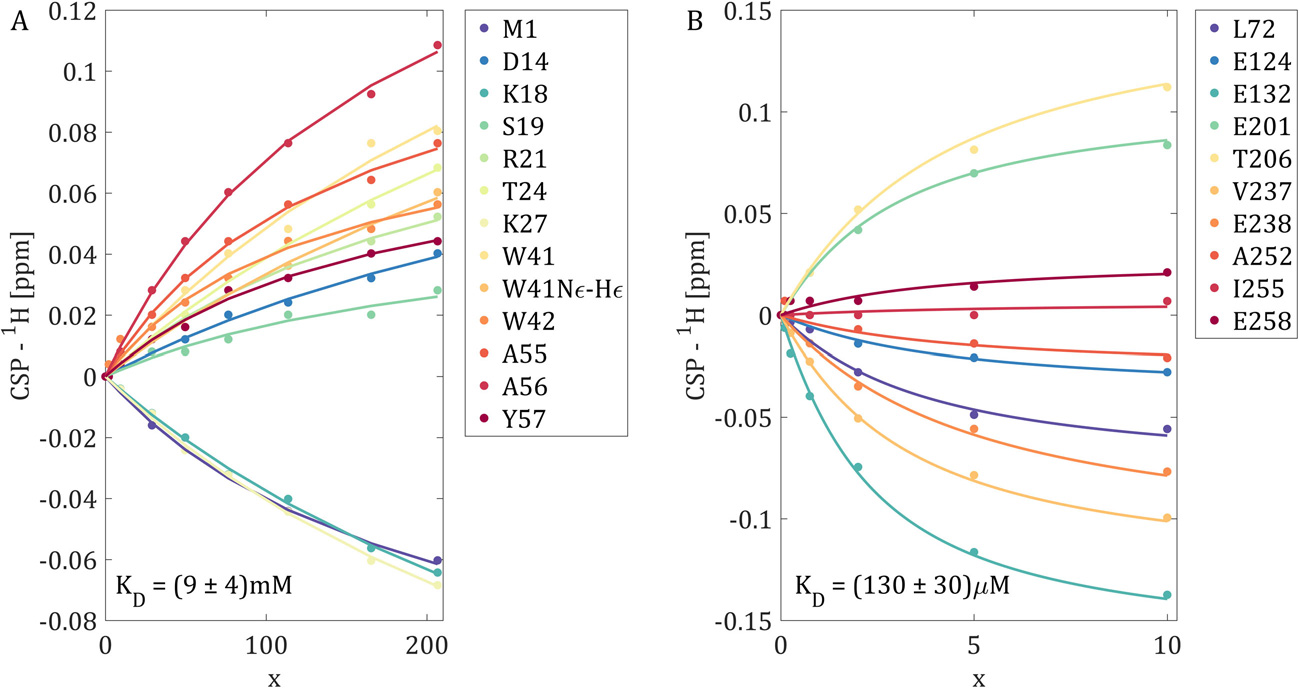
CSP (^1^H) versus ligand‐to‐protein ratio (*x*) for selected signals from 2D ^1^H–^15^N correlation spectra. A) p41 binding by SH3; B) p12 binding by PCNA. Curves were obtained by least squares data fitting to Equation (1) and using series of fully sampled 2D spectra (herein called conventional method). Final *K*_D_ values and errors are the averages and standard deviations over all selected signals, respectively.

Figure 3 shows a comparison of parameters {Δ*v*_1_,Δ*v*_2,_ *K*_D_} derived by NOSCO co‐processing vs. conventional fitting. For SH3/p41 binding, the processing of NOSCO NUS acquired time‐domain data (8⋅12.5 % sampling levels) and averaging over 13 residues yields a *K*_D_=(9±4) mM, in full agreement with the conventional method (using 100 % sampled 2D spectra). For the PCNA/p12 system, the full time‐domain data had been sampled conventionally before,^[15]^ from which we constructed a NOSCO NUS matrix by appropriate selection of *t*_1_ data points (6⋅16.7 % sampling levels). Again, the averaged (over 10 residues) *K*_D_=(130±50) μM from NOSCO processing agreed fully with the conventionally obtained result, except for an apparently larger error. PSO parameters used were: 1000 particles, 10 iterations, 5 runs (see SI). NOSCO processing took approximately 15 minutes of computation time per selected single‐peak spectral region (with 4‐fold zero filling in both dimensions separately) running on an Intel(R) Xeon(R) CPU E5‐2680 v2 @ 2.80 GHz with 48 GB of RAM and using MATLAB 2015b with Parallel Computing Toolbox.

**Figure 3:**
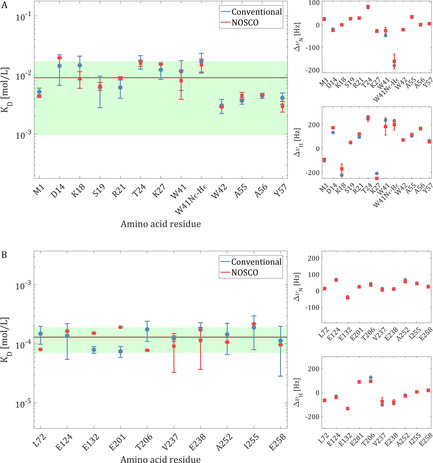
Individual *K*_D_ values (log scale) and CSP amplitudes versus residue. A) p41 binding by SH3; B) p12 binding by PCNA. Blue circles: Results from conventional fitting of individual ^1^H‐^15^N titration spectra. Red squares: Results from NOSCO co‐processing (error bars correspond to the standard deviation from 20 repeats using the same NOSCO time domain data, see Supporting Information). Left: Individual *K*_D_ values, with indication of the average value (identical for both analysis methods) and 95 % confidence interval (green band). Right: CSP amplitudes (in Hz) for ^15^N (top) and ^1^H (bottom); the indicated limits of *y* axis correspond to the specified parameter boundaries for NOSCO processing.

Figures 2 and 3 show the per residue and averaged *K*_D_ values obtained for both protein/ligand systems by conventional data analysis vs. NOSCO co‐processing, respectively. For the SH3/p41 system, 13 signals were suited for both conventional and NOSCO analysis. For the PCNA/p12 system, 22 signals showed sufficient CSP for conventional analysis,^[15]^ but high spectral crowding allowed NOSCO analysis only for 10 well resolved signals (i.e. single peak containing spectral regions), which reduced the accuracy of *K*_D_ determination by this method simply due to the fewer data for averaging. Finally, the PCNA/p12 system also shows common signal decrease and broadening during titration (see SI). In contrast to dilution, the effect of such (chemical or conformational) signal broadening is neither uniform nor known a priori and, therefore, cannot be compensated by NOSCO. Yet, our results show that NOSCO processing can provide reasonable results even in these cases.

In summary, we have presented a complementary t_1_ sampling schedule to record an entire titration series of 2D experiments as one *pseudo* 3D NOSCO NUS experiment, where the added dimension encodes the variable ligand‐to‐protein ratio in an unambiguous, traceable manner. Co‐processing of such pseudo 3D data produces a single projected 2D spectrum, where signals affected by binding induced CSP appear smeared out. These can be refocused by dedicated NOSCO processing to extract accurate values for *K*_D_ and the CSP amplitudes that are of great significance, e.g., for structure based ligand optimization. Currently, the method is limited to a single binding‐site model and 2D spectra, but can be extended to other binding models and higher dimensionality.

The overall measurement times for titration series are reduced by an order of magnitude, that is, by the number of ligand‐to‐protein ratios screened, and may be further reduced by combining NOSCO processing with CS reconstruction (see SI). This may open new and broader applications of NMR titration experiments, e.g., to unstable target proteins (or ligands) and in industrial drug discovery projects.

## Conflict of interest

The authors declare no conflict of interest.

## Supporting information

As a service to our authors and readers, this journal provides supporting information supplied by the authors. Such materials are peer reviewed and may be re‐organized for online delivery, but are not copy‐edited or typeset. Technical support issues arising from supporting information (other than missing files) should be addressed to the authors.

Supplementary

Supplementary
