# Supplementary material for: Non‐Stationary Complementary Non‐Uniform Sampling (NOSCO NUS) for Fast Acquisition of Serial 2D NMR Titration Data
Source: Angew Chem Int Ed Engl. 2020 Sep 29;59(52):23496–9. doi: 10.1002/anie.202009479 (PMC7756666; doi:10.1002/anie.202009479)
Supplement: Supplementary file 1 — Supplementary [file ANIE-59-23496-s001.pdf]

Angewandte Chemie
Eine Zeitschrift der Gesellschaft Deutscher Chemiker

Supporting Information

Non-Stationary Complementary Non-Uniform Sampling (NOSCO NUS) for Fast Acquisition of Serial 2D NMR Titration Data

*Javier A. Romero, Ewa K. Nawrocka, Alexandra Shchukina, Francisco J. Blanco, Tammo Diercks, and Krzysztof Kazimierczuk**

anie_202009479_sm_miscellaneous_information.pdf
anie_202009479_sm_PeakCorrect_Video.avi

Author Contributions

J.R. Data curation: Lead; Formal analysis: Lead; Investigation: Lead; Methodology: Equal; Software: Lead; Visualization: Lead; Writing - Original Draft: Equal
E.N. Data curation: Equal; Investigation: Supporting
A.S. Software: Equal
F.B. Conceptualization: Supporting; Data curation: Equal; Formal analysis: Supporting; Resources: Equal; Writing - Original Draft: Supporting
T.D. Data curation: Supporting; Formal analysis: Supporting; Methodology: Supporting; Supervision: Supporting; Writing - Original Draft: Equal
K.K. Formal analysis: Equal; Funding acquisition: Lead; Methodology: Equal; Project administration: Lead; Writing - Original Draft: Equal.

Experimental details

All NMR measurements for the SH3/p41 system were performed on a 700 MHz Agilent DirectDrive2 spectrometer equipped with a room-temperature HCN probe at 25 °C. A 25 μM solution of ^{15}N α-Spectrin domain SH3 in 10 mM sodium citrate buffer (pH=3.5) was purchased from Giotto Biotech (Sesto Fiorentino, Italy). The ligand decapeptide p41[1,2] with amino acid sequence APSYSPPPPP was purchased from Lipopharm.pl (Poland). A stock solution of p41 peptide was obtained by dissolving 4.8 mg of the peptide in 200 μl of a citrate buffer (pH = 3.5).

2D ^{1}H-^{15}N HSQC titration spectra were recorded with 256 t_1 increments, 8 scans, F2(^{1}H) acquisition time 0.089 s. For each ligand-to-protein ratio x, the same 2D spectrum was repeated, however, with NUS sampling of only 32 randomly selected points (but in such a way that NUS schedules are complementary and add up to a single, complete 2D HSQC signal). Each conventional and NUS 2D experiment required 94 and 12 minutes of spectrometer time, respectively. The titration series was recorded with 8 different values of x: 2.07, 9.3, 28.93, 49.59, 76.45, 113.64, 165.29, 206.61. Additionally, a reference spectrum was recorded identically prior to adding any ligand.

For the PCNA/p12 system, all 2D ^{1}H-^{15}N TROSY spectra were recorded on an 800 MHz Bruker AVANCE III spectrometer at 35 °C, as previously described.[3] The titration series comprises the reference spectrum (no ligand) and 6 with different values of x: 0.1, 0.25, 0.5, 2, 5, 10. Each experiment required 22 hours NMR time.

Reference spectra

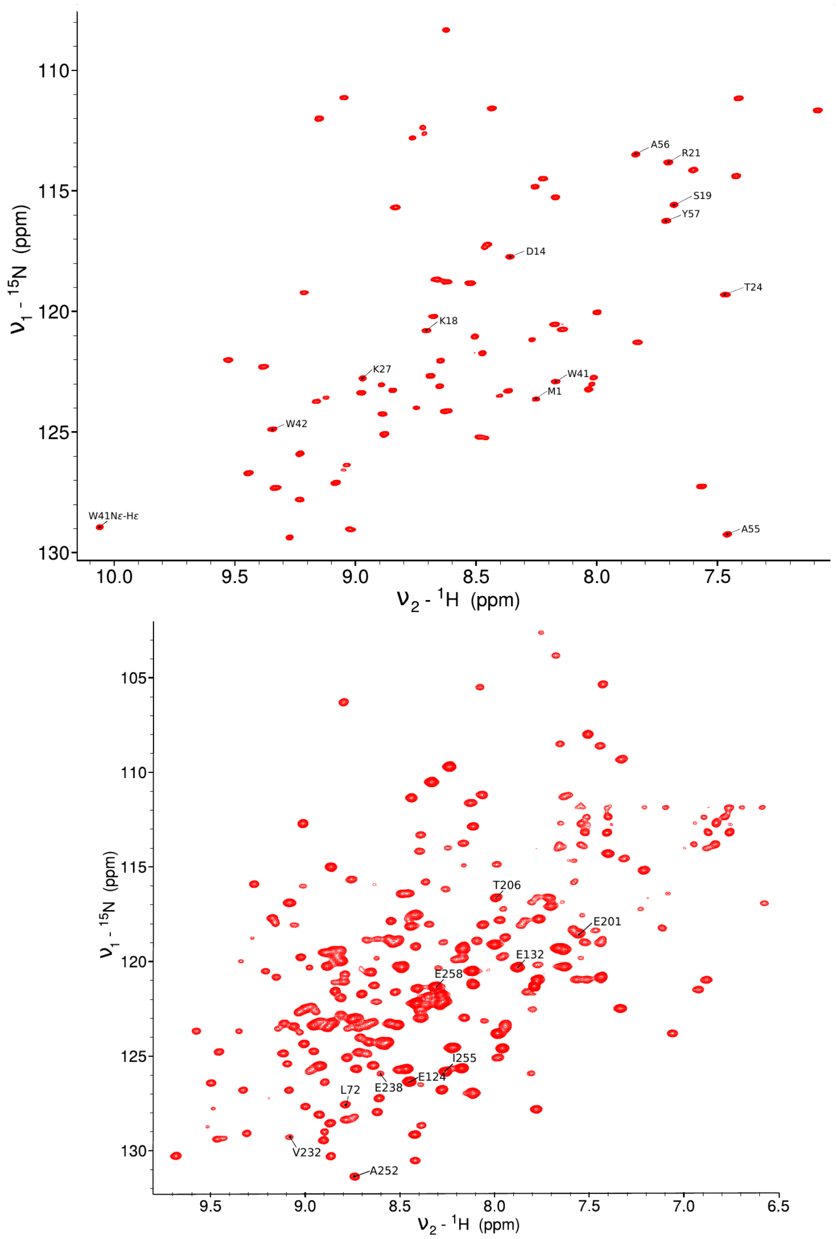

Figure S1: 2D ^{1}H—^{15}N HSQC spectrum of [U-^{15}N] labeled SH3 (upper panel) and TROSY spectrum of [U-^{2}H,^{13}C,^{15}N] labeled PCNA (lower panel). The SH3 spectrum shows significantly higher spectral sparsity, which can be defined as a number of "significant spectral points" (points contributing to peaks) in a ^{15}N dimension. The comparison explains why severe undersampling works better for the SH3 spectrum than for the PCNA spectrum.

Standard NUS reconstruction

Alternatively to NOSCO processing, one can perform a separate compressed sensing processing (NUS reconstruction) of the data collected for each titration point. However, the sampling level that is sufficient for NOSCO (e.g. 12.5% for SH3 data), might be too low when performing regular CS processing. Figure S2 shows four peaks from a series of SH3 spectra reconstructed separately from the same data as used for NOSCO. While CS performs well in the sparse spectral region (peak W41Ne-He), it would require far more sampling points to reconstruct other peaks. Problems manifest themselves in corrupted lineshapes and peak positions, up to complete disappearance of peaks.

The situation is even worse in the case of PCNA system, where obtained TROSY spectrum is much less sparse. Figure S3 shows an attempt to reconstruct one of the spectra in a titration series from the amount of data used for NOSCO ($\sim 17\%$). The reconstruction fails completely and all shifting peaks are missing.

In both cases the reconstruction has been performed using CS module of the mddnmr program (IRLS algorithm, 20 iterations).[4]

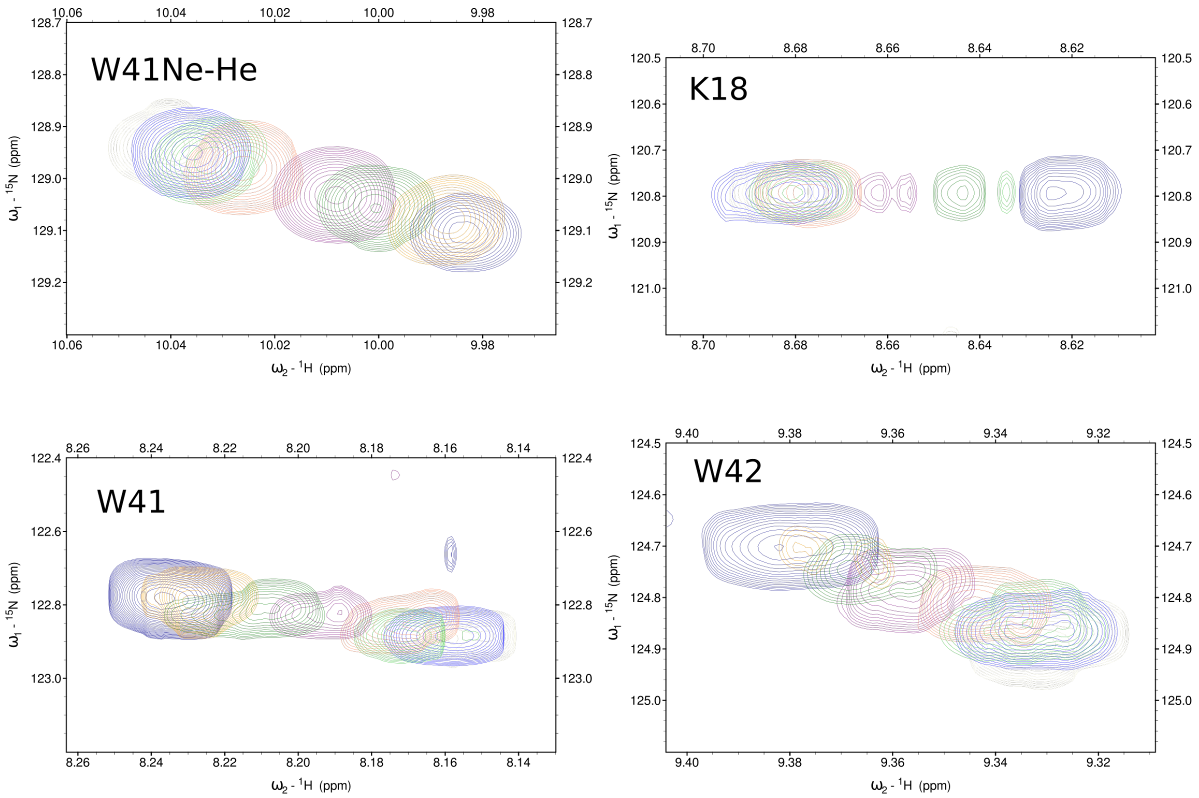

Figure S2: Selected peaks in 2D ^{1}H—^{15}N HSQC spectra of [U-^{15}N] labeled SH3 reconstructed separately from the complementary NUS data (32 points out of 256 point grid). Eight titration points (growing concentration of the ligand) correspond to colors: beige, blue, coral, lime green, purple, green, orange, dark blue. Only W41Ne-He peak belonging to the sparsest spectral region is well reconstructed using compressed sensing. Others reveal disturbed lineshapes, positions or are completely missing.

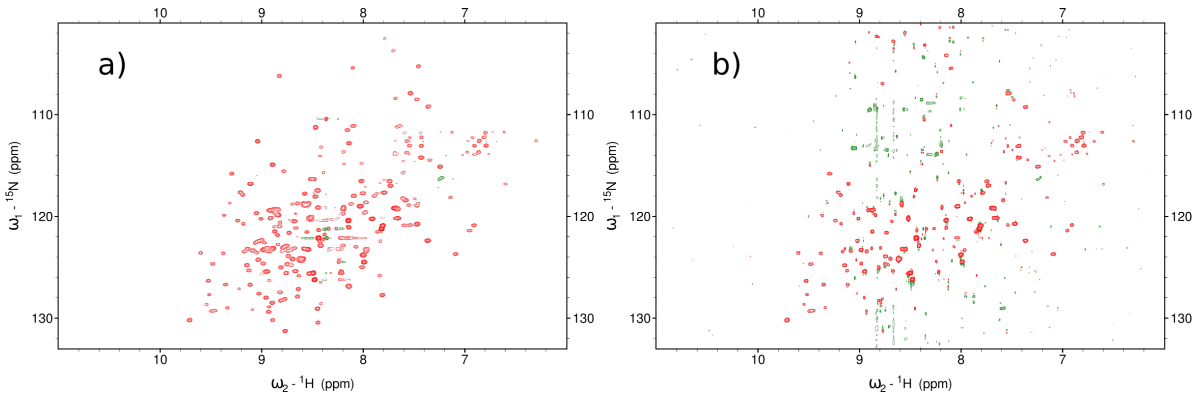

Figure S3: Fully sampled TROSY spectrum of the first titration point for PCNA (a) and an attempt to reconstruct this spectrum using compressed sensing (b) from 21 NUS points out of 128-point grid (the same as used for NOSCO processing). Besides sparse region in the left part of the spectrum, the reconstruction failed completely and all shifting peaks are missing.

NOSCO spectra

Figures S4 and S5 and illustrate the performance of NOSCO processing in refocusing peaks that appear smeared by binding induced CSP in the co-processed 2D projection spectrum from a pseudo 3D titration series sampled with NOSCO NUS. For the well resolved SH3 spectrum, NOSCO refocused and reference signals are virtually identical. In contrast, the co-processed PCNA titration series spectrum contains stronger t_1-noise artifacts resulting from non-stationarity[5] due to intrinsically higher spectral crowdedness and lower signal-to-noise ratio. While this leads to a generally poorer match between NOSCO refocused and reference peaks (which often show partial overlap), the derived K_D values still agree precisely with those obtained by the conventional method. Similar excellent agreement is observed for the CSP amplitudes derived by both methods, confirming that NOSCO processing can also reliably quantify these critical structural parameters for advanced docking methods.[6,7]

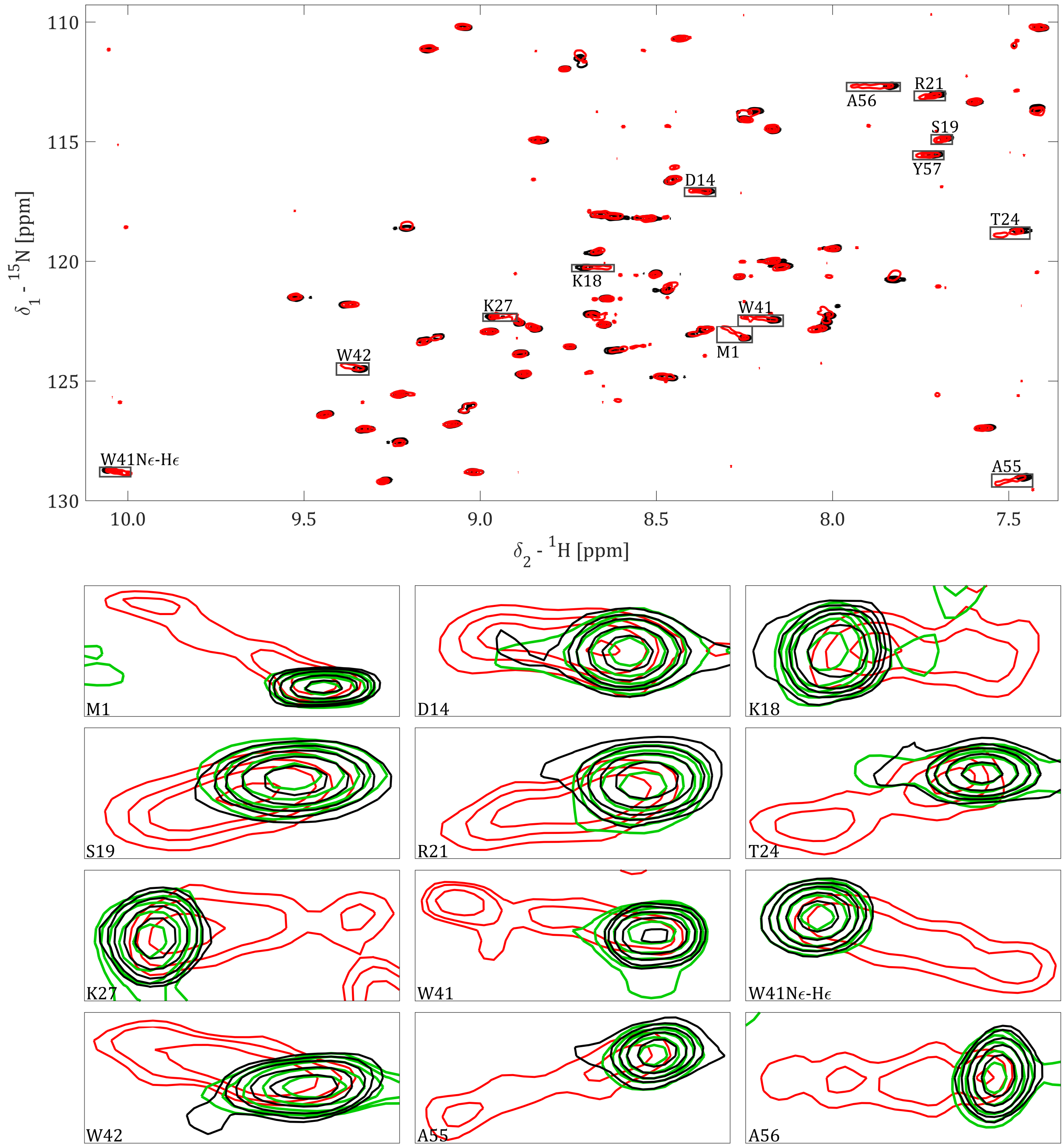

Figure S4: (Top) Superimposed 2D ^{1}H-^{15}N HSQC spectra of ligand-free SH3 (black, reference) and from co-processing of the NOSCO sampled titration series with p41 (red) where indicated signals appear smeared by binding induced CSP. (Bottom) Corresponding spectral regions containing a single smeared peak (red). Individual NOSCO processing achieves its refocusing (green) onto the corresponding reference signal (black) by adjusting the CSP amplitudes $(\Delta\nu_H, \Delta\nu_N)$ and K_D value.

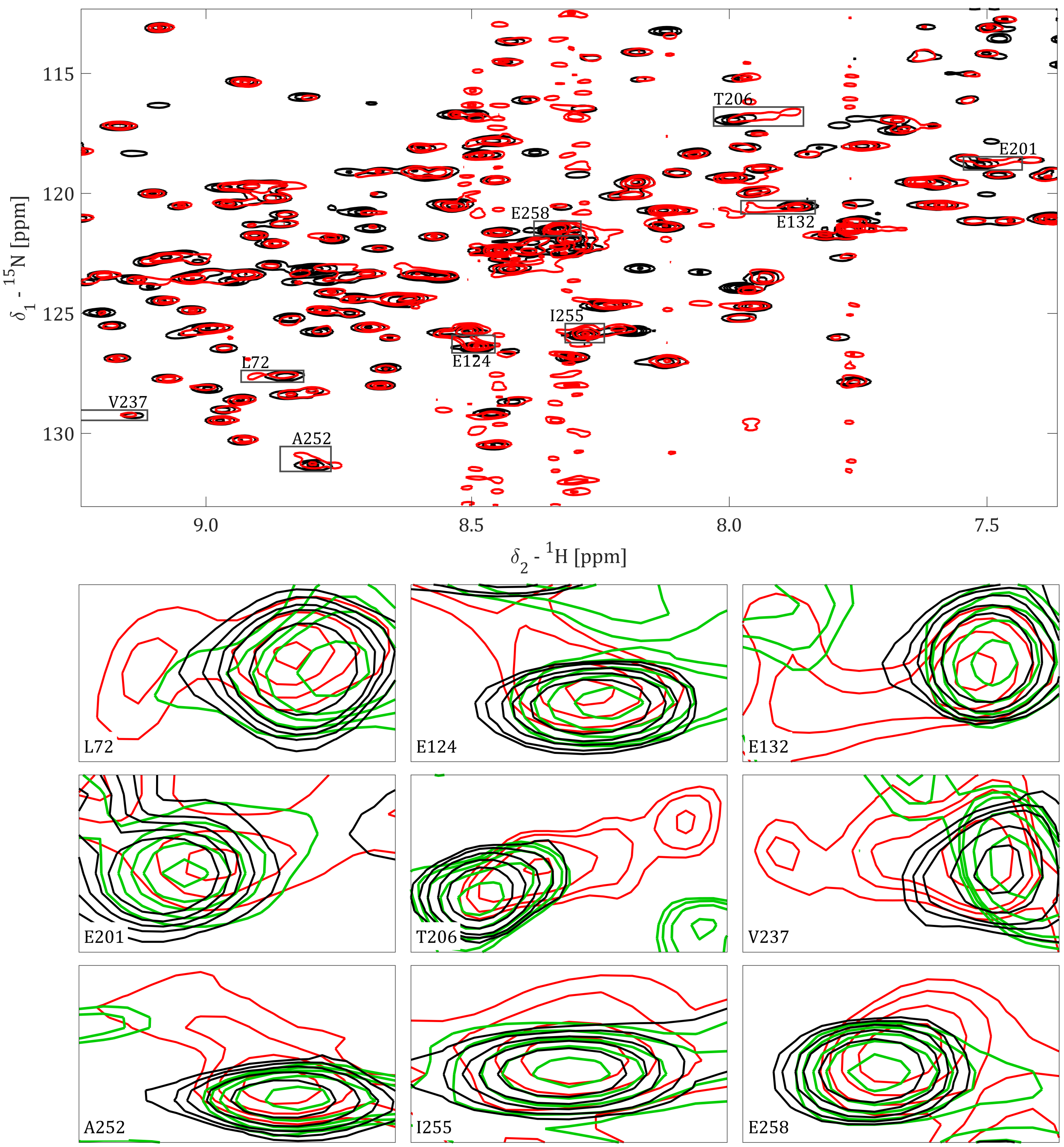

Figure S5: (Top) Superimposed 2D ^{1}H-^{15}N HSQC spectra of ligand-free PCNA (black, reference) and from coprocessing of the NOSCO sampled titration series with p12 (red) where indicated signals appear smeared by binding induced CSP. (Bottom) Corresponding spectral regions containing a single smeared peak (red). Individual NOSCO processing achieves its refocusing (green) onto the corresponding reference signal (black) by adjusting the CSP amplitudes $(\Delta\nu_H, \Delta\nu_N)$ and K_D value.

Particle swarm algorithm

Particle swarm optimization algorithm (PSO)[8] belongs to so-called evolutionary algorithms. PSO efficiently searches for a global extreme and can deal with functions with many local extremes. A sufficient number of particles (points of the function domain) allows avoiding the fall into a local extreme. PSO is also suitable for non-smooth functions as it does not use the gradient of the sought-for function. The algorithm is initialized with picking up random points, or particles, within the function domain (the user-provided constraints). For each of these candidate solutions to the problem, the function value is calculated. At each iteration step, the positions of all particles are updated, with a stochastic and a deterministic component. The particle "velocities" are calculated as follows:

$$\begin{aligned} v_j^{i+1} = wv_j^i + c_1 r_1 (xBest_j^i - x_j^i) + c_2 r_2 (gBest_j^i - x_j^i) \\ x_j^{i+1} = x_j^i + v_j^i \end{aligned} \tag{1}$$

where i is the number of the iteration, j is the index of the particle, x is the particle position in the multidimensional space of search, v is its velocity, w, c_1 and c_2 are hyperparameters of the algorithm ("inertia" and "acceleration" factors), r_1 and r_2 are random numbers between 0 and 1, $xBest$ and $gBest$ are the best particle position and the best group position. Thus, some kind of group behaviour also governs the displacement of every particle. In this work, we adjusted the MATLAB code provided by L.A. Kareem[9] and tuned the hyperparameters. Here, particle velocities are reversed if particles attempt going out of the search space defined by the user. Moreover the algorithm performs several runs with independent initializations and selects the best result. This is desirable because of the semi-stochastic nature of the algorithm.

A brute force search for the set of parameters $\{\Delta\nu_N, \Delta\nu_H, K_D\}$ that minimize Eq. 4 (in the main text) requires the pre-definition of search boundaries. Boundaries for $\Delta\nu$ values can be estimated by looking at the size of "smeared" peaks in the complementary NUS

spectrum (see Figure 1 in main text). Yet, an underestimation results if the CSP do not reach their plateau (saturation) value by the end of the titration, which is the case for both systems studied in this work, and more severely so for the SH3/p41 system. The problem can be alleviated by reasonable up-scaling of the estimations, suggesting CSP amplitudes of 0.5 ppm and 3 ppm in the ^{1}H and ^{15}N dimensions, respectively (in agreement with typical $\Delta\nu$ values[6]). NOSCO automatically sets boundary values for K_D using Eq. 2 (in the main text). The lower boundary is given by the K_D value that results in a 1% difference for the CSP values of the last two titration points in the series. The upper boundary is then set three orders of magnitude higher. Such a wide range of K_D values might seem exaggerated, but helps to avoid incorrect results without an excessive increase in computation time.

Working principle of NOSCO: animation

As further SI material we include an animation illustrating how NOSCO works. This simulation was performed with hyperparameters for Particle Swarm Optimization different from those described in the main text.

The left-hand panel shows the 3-dimensional parameter space in which the PSO search is carried out. The entire sequence corresponds to a single PSO run. The red particle is the best particle after each iteration. The two grey particles are bound to the ν_1 (^{15}N) vs ν_2 (^{1}H) and ν_1 (^{15}N) vs K_D limits and show the projections of the best particle onto these walls. Green lines mark the true parameter values obtained in this search.

The central panel is a surface plot showing how the complementary NUS signal is corrected by multiplication with a correction signal fed with the parameters from the best particle in each iteration.

The right-hand panel shows contour plots of NOSCO sampled, reference, and corrected peaks.

The sub-optimal results obtained in the animation are due to the alteration of PSO hyperparameters (reduced number of particles and convergence). Yet, this example helps to visualize the complexity of the problem, where many possible solutions (local minima) yield very similar results (as seen in this simulation) and only the corrected peak height is satisfactorily reproduced. At the end of the animation, each blue particle not positioned with the bulk of particles is also trapped in a local minimum.

Sampling multidimensional NMR signals

NMR frequency sampling in an indirect time dimension is a lengthy process governed by three fundamental relations: (i) the time increment ($\Delta\tau$) between sampling points inversely defines the spectral width ($SW = 1/\Delta\tau$) for unaliased frequency detection (Nyquist theorem[10]), (ii) the longest sampling time (acquisition time AQ) inversely defines the spectral resolution ($\Delta\nu = 1/AQ$),[11] and (iii) NMR frequencies from spin rotation require pairwise sampling of the real and imaginary parts for each increment. Conventionally, AQ is reached after *uniform* sampling of all n time increments ($AQ = n \cdot \Delta\tau$), leading to long 2D vs. 1D experiment times, $T_{2D}/T_{1D} \sim 2 \cdot n = 2 \cdot (SW/\Delta\nu)$. Alternatively, AQ may be reached more quickly with fast sampling[12] and *non-uniform sampling* (NUS)[13] schemes. For typical 2D protein spectra, however, the acceleration factor is limited to between 2 (50% NUS) to 4 (25% NUS), depending on the number of peaks,[14] since each spectrum must have sufficient sensitivity and resolution to allow peak localization. Consequently, even NUS acquisition of a full 2D titration series may still take up to a week and, thus, exceed the protein's (or ligand's) lifetime or available spectrometer time. Alternative sampling and processing methods for serial experiments[15] have not yet been adapted to this application.

Undersampling and NOSCO

Of note, the complementary sampling of titration spectra does not have to cover the complete t_1 grid since NOSCO NUS can be combined with compressed sensing (CS) reconstruction.[16,17] In this case, NOSCO processing first corrects the measured signals for non-stationarity, then reconstructs the missing time-domain data points of a signal with an iterative soft thresholding algorithm[16,18] and, finally, implements the minimization procedure from Eq. (4). This *undersampling* mode is computationally more demanding and requires the spectrum to be compressible.[14]

Figure S6 shows the results of such an approach, averaged over 5 runs using the same

sampling schedule. In both cases, a total sampling level of only 12.5% was used. For the SH3/p41 sample, this implies measuring only 32 (of 256) t_1 increments, i.e. just 4 increments per titration point, resulting in very short 12 minutes for recording the entire pseudo-3D titration spectrum. For the PCNA/p12 sample, a 12.5% sampling level corresponds to a total of 16 (of 128) t_1 increments, or 3 increments per titration point, which would require less than 3 hours of total experiment time. Computation times were approximately 20 and 60 minutes per peak for PCNA/p12 and SH3/p41 samples, respectively (with machine and PSO parameters described in main text). The difference in computation times are mainly given by the t_1 grid size.

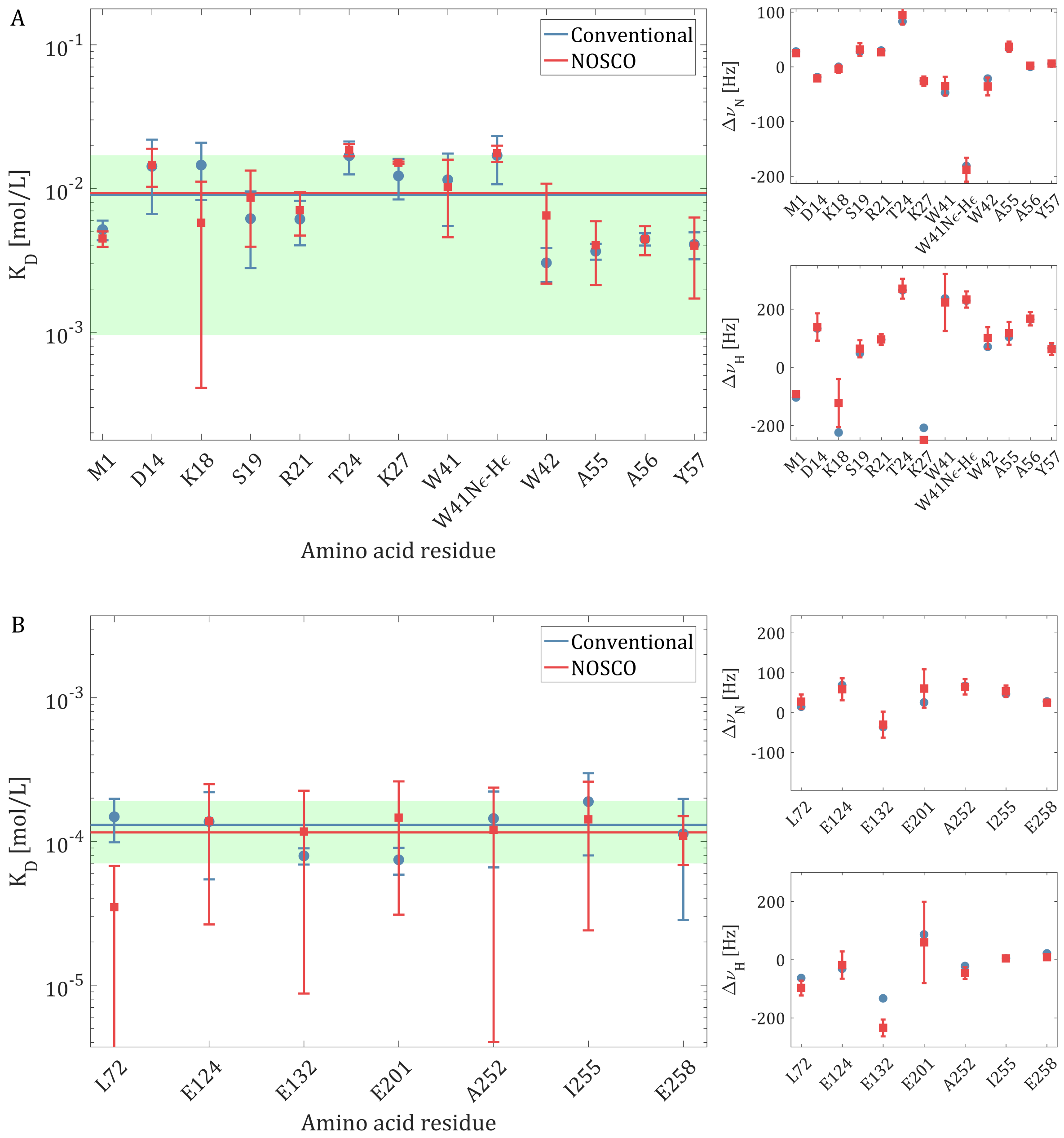

Figure S6: Results from NOSCO processing of undersampled NOSCO-NUS data. A total sampling level of 12.5% was used in both cases. (A) SH3/p41 system with a total of 32 t_1 increments, i.e. 4 increments per titration point. The final NOSCO estimation yields $K_d = (9 \pm 4)mM$. (B) PCNA/p12 system with a total of 16 t_1 increments, i.e. 3 increments per titration point. The final NOSCO estimation yields $K_d = (116 \pm 40)\mu M$ (note that 3 values were removed as outliers). The values shown are averages over 5 NOSCO iterations using the same sampling schedule; the error bars correspond to one standard deviation. PSO parameters used are the same as described in the main text. The panels on the right show the corresponding estimates for the CSP amplitudes.

Signal intensity decay

Signal intensity decay due to a transverse relaxation increasing with the ligand-to-protein ratio x is a commonly observed phenomenon in titration studies. The dependence of the net transverse relaxation rate, R_2, on x is typically distinct for each residue and cannot be assumed to be linear, complicating its consideration in the correction algorithm. Yet, our results (presented in the article) show that NOSCO processing returns accurate values for K_D and CSP that agree excellently with those obtained by the conventional approach, without any need for further adjustments. Signal intensity decay is particularly strong for the PCNA/p12 system studied here, as shown below for all selected peaks.

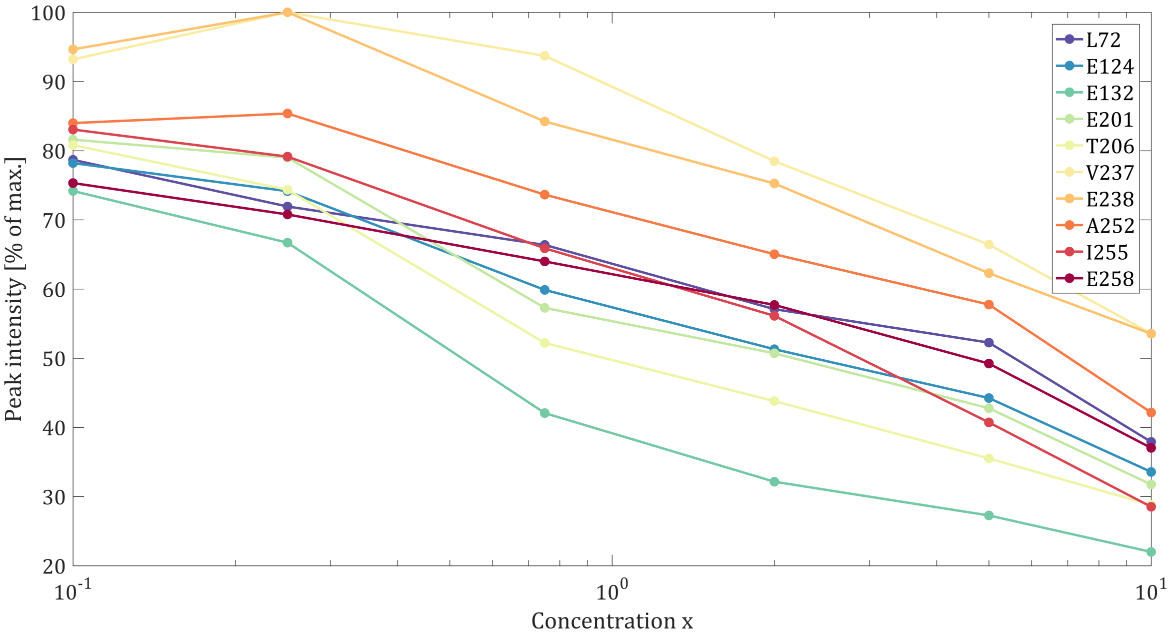

Figure S7: Signal amplitude (in % of the maximum) vs. ligand-to-protein ratio x (log scale) for all selected 10 peaks of the PCNA/p12 system, as derived from the conventionally, fully sampled 2D titration spectra of PCNA. While the signal intensity can fall below 30% of the reference peak intensity, NOSCO still returns accurate results (see main text).

Separate NOSCO runs from Fig. 3 in the main text

In Figure 3 of the main text, we show the results from NOSCO processing of NOSCO-NUS sampled titration data. These results are the average over 20 NOSCO repeats, while error bars represent one standard deviation over these 20 NOSCO repeats. While the repeats evinced the accuracy and reproducibility of NOSCO processing, exact K_D values were already obtained after a single run. In each repeat, the same optimized PSO parameters were used (see main text).

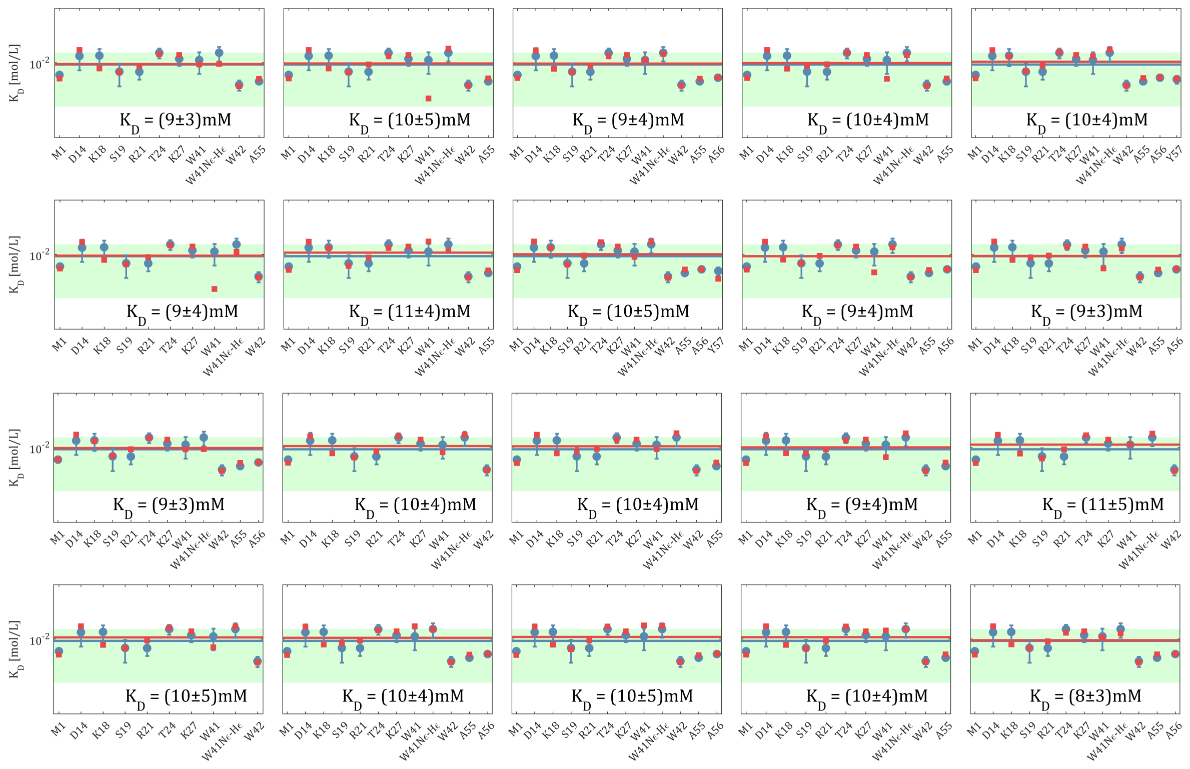

Figure S8: Results of all 20 NOSCO processing runs (on the same NOSCO-NUS t_1 data matrix) for the SH3/p41 system; the averaged values over all 20 runs are shown in Figure 3A of the main text. Blue dots and red squares represent values from conventional fitting and NOSCO processing, respectively. The green band represents the 95% confidence interval for the average K_D obtained by conventional fitting. The indicated K_D values are the average (over all selected residues) from the pertaining NOSCO processing run.

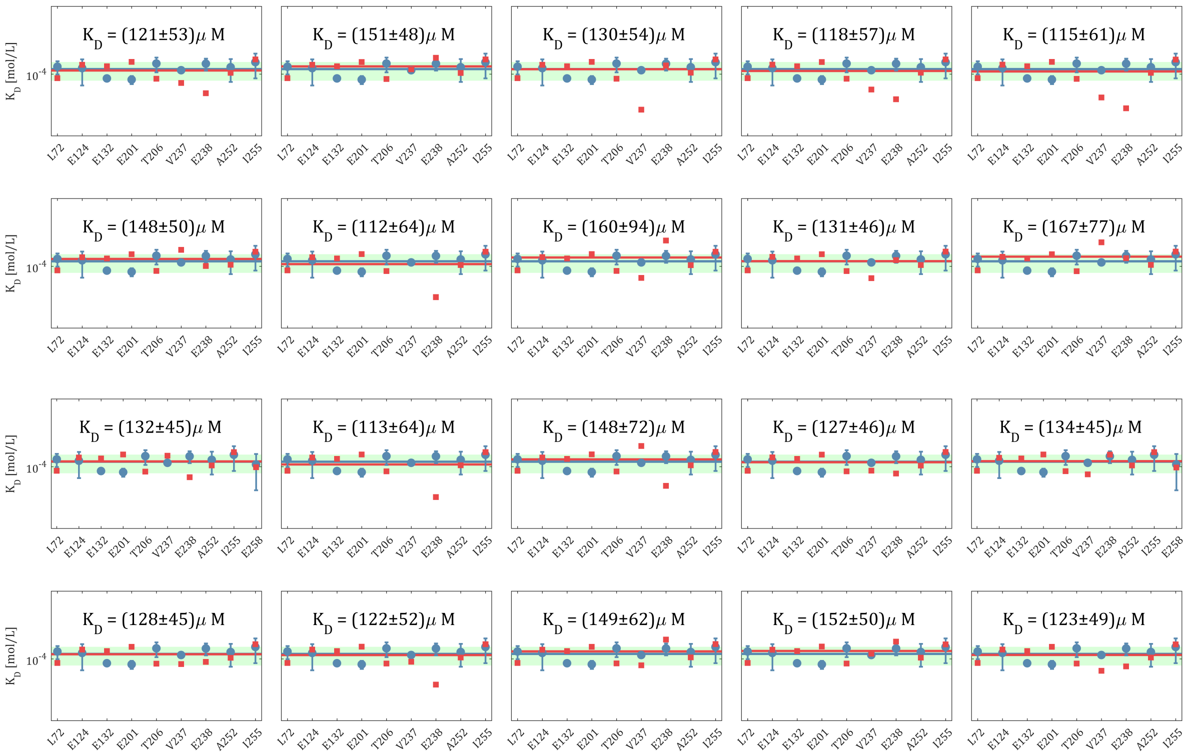

Figure S9: Results of all 20 NOSCO processing runs (on the same NOSCO-NUS t_1 data matrix) for the PCNA/p12 system; the averaged values over all 20 runs are shown in Figure 3A of the main text. Blue dots and red squares represent values from conventional fitting and NOSCO processing, respectively. The green band represents the 95% confidence interval for the average K_D obtained by conventional fitting. The indicated K_D values are the average (over all selected residues) from the pertaining NOSCO processing run.

NUS schedules generator

Here we show a short Matlab script to produce stratified NUS schedules. The output files are *.txt* files ready to be used for measurement in the NMR spectrometer. There are as many files as titration points to be studied (input by user). The NUS schedules are not completely random, but stratified, to ensure a more homogeneous sampling across each schedule.

```
% Scipt to produce stratified NUS schedules
% Output files are names "schedule_x.txt" where x is schedule number


sz1 = 130;  % Size of grid in t1 dimension
N = 8;      % Number of signals (titration points)


pts = floor(sz1/N);
lop = rem(sz1,N);
y = reshape(1:sz1-lop,[N pts]);
NUS_schedule = zeros(size(y));
for i = 1:pts
    NUS_schedule(:,i) = y(randperm(N),i);
end
ylop = sz1-lop+1:sz1;
v = [ylop(randperm(lop)) NaN(1,N-lop)]';
NUS_schedule(:,end+1) = v;
NUS_schedule = NUS_schedule-1;


for i = 1:size(NUS_schedule,1)
    name = ['schedule_',int2str(i),'.txt'];
    file = fopen(name,'w');
```

```
    aux = NUS_schedule(i,~isnan(NUS_schedule(i,:)));
    for j = 1:numel(aux)
        fprintf(file,'%d\n',aux(j));
    end
    fclose(file);
end
```

Experimental setup

The data for NOSCO processing can be collected using default NUS data collection scheme available on spectrometers of all main vendors. The only difference is that sampling schedules in the consecutive spectra should be complementary i.e. generated with the code above. Thus, after setting up NUS mode, but before running an experiment, the schedule generated by the spectrometer-driving software has to be replaced by the ”complementary” one.

Data pre-processing

The data for NOSCO should be preprocessed using nmrPipe.[19] The data has to be converted into the nmrPipe format, processed by applying zero filling, apodization and phasing in both dimensions, as well as solvent suppression. Scripts are available from `nmr.cent.uw.edu.pl` → `Downloads`. The NOSCO program compensates the signal intensity changes between NUS points that result from the dilution of a sample. It requires manual setting of the regions of interest for each peak.

nmrPipe script for pre-processing of (full) PCNA/p12 data:

```
bruk2pipe -in ./ser \
-bad 0.0 -noswap -DMX -decim 2080 -dspfvs 21 -grpdly 76 \
-xN       1024       -yN     256 \
```

```
-xT       512       -yT 128        \
-xMODE    Complex   -yMODE Echo-AntiEcho \
-xSW      9615.385   -ySW   2590.674   \
-xOBS     800.130   -yOBS   81.076     \
-xCAR     8.300 -yCAR   117.000   \
-xLAB     HN -yLAB   15N   \
-ndim  2 -aq2D States \
-out ./test.fid -verb -ov


nmrPipe -in test.fid \
| nmrPipe -fn SP -off 0.5 -end 1.00 -pow 1 -c 0.5   \
| nmrPipe -fn ZF -zf 2                               \
| nmrPipe -fn FT -auto                               \
| nmrPipe -fn CS -ls 3.6ppm -sw    \
| nmrPipe -fn FT -inv                                \
| nmrPipe -fn SOL                                    \
| nmrPipe -fn FT -auto                               \
| nmrPipe -fn CS -rs 3.6ppm -sw    \
| nmrPipe -fn PS -p0 -10.00 -p1 0.00  -verb          \
| nmrPipe -fn POLY -auto -xn 5.0ppm -ord 1           \
| nmrPipe -fn EXT -x1 10ppm -xn 6.3ppm -sw           \
| nmrPipe -fn FT -inv -neg                               \
| nmrPipe -fn TP -hyper                              \
| nmrPipe -fn SP -off 0.5 -end 1.00 -pow 1 -c 1.0   \
| nmrPipe -fn ZF -zf 2                               \
| nmrPipe -fn FT                                     \
| nmrPipe -fn PS -p0 -180.00 -p1 180.00  -verb       \
```

```
| nmrPipe -fn FT -inv                              \
   -ov -out 0.fid
```

nmrPipe script for pre-processing of (full) SH3 data :

```
#!/bin/csh

var2pipe -in ./fid \
 -noaswap  \
  -xN              2048  -yN             512  \
  -xT              1024  -yT             256  \
  -xMODE        Complex  -yMODE    Rance-Kay  \
  -xSW        11467.890  -ySW       2500.000  \
  -xOBS         700.101  -yOBS        70.949  \
  -xCAR           4.773  -yCAR       120.135  \
  -xLAB              HN  -yLAB           N15  \
  -ndim               2  -aq2D       Complex  \
  -out ./test.fid -verb -ov

  nmrPipe -in test.fid \
| nmrPipe -fn SOL                                   \
| nmrPipe -fn SP -off 0.5 -end 1.00 -pow 1 -c 0.5    \
| nmrPipe -fn ZF -zf 2                               \
| nmrPipe -fn FT -auto                              \
| nmrPipe -fn PS -p0 168.00 -p1 0.00 -verb          \
| nmrPipe -fn EXT -x1 701  -xn 1500 -sw              \
| nmrPipe -fn FT -inv                               \
| nmrPipe -fn TP -hyper                             \
```

```
| nmrPipe -fn SP -off 0.5 -end 1.00 -pow 1 -c 0.5    \
| nmrPipe -fn ZF -zf 2                                \
   -ov -out 0.fid
```

nmrPipe script for pre-processing of (NOSCO NUS) SH3 data :

```
#!/bin/csh

nusExpand.tcl -mode varian -yT 256 -sampleCount 32 \
 -in ./fid -out ./fid_full -sample ./sampling.sch

var2pipe -in ./fid_full \
 -noaswap  \
  -xN              2048  -yN              512  \
  -xT              1024  -yT              256  \
  -xMODE        Complex  -yMODE     Rance-Kay  \
  -xSW        11467.890  -ySW        2500.000  \
  -xOBS         700.101  -yOBS         70.949  \
  -xCAR           4.773  -yCAR        120.135  \
  -xLAB              1H  -yLAB            N15  \
  -ndim               2  -aq2D        Complex  \
  -out ./test.fid -verb -ov

nmrPipe -in test.fid \
| nmrPipe  -fn SOL                                    \
| nmrPipe  -fn SP -off 0.5 -end 1.00 -pow 1 -c 0.5    \
| nmrPipe  -fn ZF -zf 2                               \
| nmrPipe  -fn FT -auto                               \
```

```
| nmrPipe  -fn PS -p0 168.00 -p1 0.00 -verb           \
| nmrPipe -fn EXT -x1 701  -xn 1500 -sw               \
| nmrPipe  -fn FT -inv                                \
| nmrPipe  -fn TP -hyper                              \
| nmrPipe  -fn SP -off 0.5 -end 1.00 -pow 1 -c 0.5    \
| nmrPipe  -fn ZF -zf 2                               \
   -ov -out 1.fid
```

NOSCO: Matlab code

This is the executable Matlab script to initialize the processing method.

```
%% NOSCO


%%%%%%%%%%%%%%%%%%%%%%%%% OPTIMIZATION PARAMETERS %%%%%%%%%%%%%%%%%%%%%%%%%
N = 1000;           % Number of particles to run PSO
maxite = 10;        % Number of iterations for PSO
maxrun = 5;         % Number of runs for PSO
tol = 0;            % Tolerance
OptParam = {N,maxite,maxrun,tol};
%%%%%%%%%%%%%%%%%%%%%%%%%%%%%%%%%%%%%%%%%%%%%%%%%%%%%%%%%%%%%%%%%%%%%%%%%%%


% Folder path
addpath('NMRData')


res = NOSCO(@Sampling,OptParam);
```

Sampling is a function with no input and 2 cell arrays as output. The script within this function is different depending on whether data was acquired using a complementary NUS schedule or the complementary NUS signal will be artificially generated using fully sampled signals. Here we show the former case.

```
function [ExpData,NUSData] = Sampling()
%%%%%%%%%%%%%%%%%%%%%%%%%%%%%% INPUT VALUES %%%%%%%%%%%%%%%%%%%%%%%%%%%%%%%
% Experimental parameters
sample = 'SH3';         % Sample's name, tag or label
SF = 700;               % Spectrometer frequency in MHz
```

```
sz1 = 256;              % Data points in indirect dimension
sz2 = 200;              % Data points in direct dimension
SW1 = 2500.00;          % Spectral Width in indirect dimension
SW2 = 2239.822;         % Spectral Width in direct dimension
zff1 = 4;               % Zero-filling factor in dimension 1 (indirect)
zff2 = 4;               % Zero-filling factor in dimension 2 (direct)
files = 0:8;            % Files for processing
G0 = 21.12e-6;          % Initial protein concentration [mol/L]
% Concentrations (ligand:protein)
R = [2.07 9.3 28.93 49.59 76.45 113.64 165.29 206.61];
% Dilution compensation factors
D = [1.0018 1.0082 1.0255 1.0436 1.0673 1.1 1.1455 1.1818];
G = G0./D;


% Coordinates of maxima of mother spectrum for selected peaks
% At the end of this function there is a script that
% can be used to plot spectra and find these values.
%       D1  D2
cenpts=[593 302;
        651 596;
        400 188;
        778 125;
        615 323;
        589 503;
        330 186;
        320 219;
        489 127;
```

```
        380 180;
        443 350;
        769 775;
        532 436];
% Zero filling factors used for recording cenpts values
        zff1cp = 4;
        zff2cp = 4;
%%%%%%%%%%%%%%%%%%%%%%%%%%%%%%%%%%%%%%%%%%%%%%%%%%%%%%%%%%%%%%%%%%%%%%%%%%%%%

% PRE-PROCESS ------------------------------------------------------------%
SF1 = (sz1/(sz1-1))*SW1;    % Sampling frequency t1
SF2 = (sz2/(sz2-1))*SW2;    % Sampling frequency t2

% Adjust center coordinates of peaks according to zero filling factors.
cenpts = round([cenpts(:,1)*(zff1/zff1cp) cenpts(:,2)*(zff2/zff2cp)]);
ws1 = round(1.2*zff1);      % windows size 1
ws2 = round(1.2*zff2);      % windows size 2

% Create window location of peaks
winloc(:,1) = cenpts(:,1)-repmat(ws1,size(cenpts,1),1);
winloc(:,2) = cenpts(:,1)+repmat(ws1,size(cenpts,1),1);
winloc(:,3) = cenpts(:,2)-repmat(ws2,size(cenpts,1),1);
winloc(:,4) = cenpts(:,2)+repmat(ws2,size(cenpts,1),1);
% VALIDATION -------------------------------------------------------------%

% minimal check
narginchk(0,0)
```

```
nargoutchk(2,2)

% Validate experimental data
validateattributes(sz1,{'numeric'},{'>',0,'real','scalar'});
validateattributes(sz2,{'numeric'},{'>',0,'real','scalar'});
validateattributes(SW1,{'numeric'},{'>',0,'real'});
validateattributes(SW2,{'numeric'},{'>',0,'real'});
validateattributes(zff1,{'numeric'},{'>=',1,'real','scalar'});
validateattributes(zff2,{'numeric'},{'>=',1,'real','scalar'});
validateattributes(files,{'numeric'},{'row','>=',0,'increasing'});
validateattributes(G,{'numeric'},{'row','>',0,'decreasing'});
validateattributes(R,{'numeric'},{'row','>',0,'increasing'});
validateattributes(D,{'numeric'},{'row','>',1,'increasing'});
% SAMPLING ---------------------------------------------------------------%

% Basic definitions and preallocation
Nfiles = length(files);
fid = zeros(2*sz1*zff1,2*sz2*zff2,Nfiles);
nuslist = zeros(sz1/(Nfiles-1),Nfiles-1);
R_scheme = zeros(sz1,1);
G_scheme = zeros(sz1,1);

for i = 1:Nfiles
    file = fopen([int2str(files(i)),'.fid']);
    aux = fread(file,'single');
    if(i == 1)
        fid(:,:,i) = reshape(aux(513:end),2*sz1*zff1,2*sz2*zff2);
```

```
    else
        fid(:,:,i) = reshape(aux(513:end),2*sz1*zff1,2*sz2*zff2).*D(i-1);
        nuslist(:,i-1) = dlmread(['nus',int2str(files(i)),'.txt'])+1;
        R_scheme(nuslist(:,i-1),1) = R(i-1);
        G_scheme(nuslist(:,i-1),1) = G(i-1);
    end
    fclose(file);
end

data = fid(1:end/2,:,:)+1i*fid(end/2+1:end,:,:);
fidmo = real(fftshift(fft(data,sz1*zff1,1),1));
fidmocplx = fidmo(:,1:2:end,:)+1i*fidmo(:,2:2:end,:);
spectra = real(fftshift(fft(fidmocplx,sz2*zff2,2),2));
NUS_signal = squeeze(data(1:sz1,:,2));

% Estimation of CIS values boundaries (input by user)
amps = dlmread('CIS_boundaries.txt');

% Cell arrays with data
ExpData = {sample,SF,[sz1 sz2],[zff1 zff2],[SF1 SF2],G,R,cenpts,winloc};
NUSData = {NUS_signal,R_scheme,G_scheme,spectra,amps};
%%%%%%%%%%%%%%%%%%%%%%%%%%%% PLOTTING SCRIPT %%%%%%%%%%%%%%%%%%%%%%%%%%%%%%%

% PLOT NUS AND MOTHER SPECTRA --------------------------------------------%
% Nfiles = length(files);
% fid = zeros(2*sz1*zff1,2*sz2*zff2,Nfiles);
% nuslist = zeros(sz1/(Nfiles-1),Nfiles-1);
```

```
%
% for i = 1:Nfiles
%     file = fopen([int2str(files(i)),'.fid']);
%     aux = fread(file,'single');
%     if(i == 1)
%         fid(:,:,i) = reshape(aux(513:end),2*sz1*zff1,2*sz2*zff2);
%     else
%         fid(:,:,i) = reshape(aux(513:end),2*sz1*zff1,2*sz2*zff2).*D(i-1);
%         nuslist(:,i-1) = dlmread(['nus',int2str(files(i)),'.txt'])+1;
%     end
%     fclose(file);
% end

% data = fid(1:end/2,:,:)+1i*fid(end/2+1:end,:,:);
% fidmo = real(fftshift(fft(data,sz1*zff1,1),1));
% fidmocplx = fidmo(:,1:2:end,:)+1i*fidmo(:,2:2:end,:);
% spectra = real(fftshift(fft(fidmocplx,sz2*zff2,2),2));
%
% M = max(max(spectra(:,:,1)));
% v = [0.8 0.6 0.4 0.2 0.1]*M;
% figure()
% contour(spectra(:,:,1),v,'k','LineWidth',2),hold on
% contour(spectra(:,:,2),v,'r','LineWidth',2)
%
% for i = 1:size(winloc,1)
%     rectangle('Position',[winloc(i,3) winloc(i,1)...
%         winloc(i,4)-winloc(i,3) winloc(i,2)-winloc(i,1)],'LineWidth',2);
```

```
% end
end
```

NOSCO is the main function which performs K_d boundary estimations, peak correction for all peaks selected, post-processing and plotting of results. Output from *NOSCO* is a structure array with all meaningful information from data and processing.

```
function res = NOSCO(Sampling,OptParam,varargin)
% Non-Stationary COmplementary NUS spectroscopy method for determination
% of dissociation constants (Kd) in protein-ligand complex using a single
% site binding model.
%
% Usage:
%   NOSCO(Sampling,OptParams)
%   NOSCO(Sampling,OptParams,name,value)
%
% Input:
%   Sampling -- A function, specified using @, that has no arguments and
%               that returns the cell arrays ExpData and NUSData.
%   OptParam -- Cell array containing optimization parameters defined in
%               RunMe script.
%
% Optional input:
%   varargin -- name/value pairs
%   'v0',v0 -- initial velocity
%   'c1',c1 -- pbest acceleration factor
%   'c2',c2 -- gbest acceleration factor
%   'wmax',wmax -- maximum inertia weight
%   'wmin',wmin -- minimum inertia weight
```

```
%   'pngres',pngres -- set png resolution (def '-r200')
%   'Kr',[Kmin Kmax] -- range for searching Kd (default: automatically)
%
% Optional output:
%   res -- structure with results
%   res.sample -- sample name
%   res.estimations -- matrix with NOSCO estimation values
%   res.signal -- NUS signal
%   res.Rscheme -- ligand:protein concentrations from NUS schedule
%   res.Gscheme -- total protein concentrations from NUS schedule
%   res.spectra -- mother and NUS spectra
%   res.CISamplitudes -- CIS amplitudes used to estimate CIS values
%   res.Nparticles -- number of particles used in PSO
%   res.maxite -- iterations used in PSO
%   res.maxrun -- run used in PSO
%   res.tolerance -- tolerance provided for PSO
%   res.Krange -- range used to look for K values (in order of magnitude)
%   res.v0 -- initial particles velocity
%   res.c1 -- acceleration factor 1
%   res.c2 -- acceleration factor 2
%   res.wmax -- maximum weighing factor
%   res.wmin -- minimum weighing factor
%-----------------------------------------------------------------------%

    % minimal check
    narginchk(2,16)
    nargoutchk(0,1)
```

```
% Default varargin values
v0 = 0.1;           % Initial velocity
c1 = 0.4;           % Acceleration factor
c2 = 1;             % Acceleration factor
wmax = 0.9;         % Inertia weight
wmin = 0.3;         % Inertia weight
pngres = '-r200';   % plot resolution
Kr = [];            % Kd range

if ~isempty(varargin)
    for n = 1:2:length(varargin)
        switch lower(varargin{n})
            case 'v0'
                % set particles initial velocity
                v0 = varargin{n+1};
                validateattributes(v0,{'numeric'},{'real'});
            case 'c1'
                % set pbest acceleration factor
                c1 = varargin{n+1};
                validateattributes(c1,{'numeric'},{'real'});
            case 'c2'
                % set gbest acceleration factor
                c2 = varargin{n+1};
                validateattributes(c2,{'numeric'},{'real'});
            case 'wmax'
                % set maximum inertia weight
```

```
                wmax = varargin{n+1};
                validateattributes(wmax,{'numeric'},{'>=',0,'<=',1});
            case 'wmin'
                % set minimum inertia weight
                wmin = varargin{n+1};
                validateattributes(wmax,{'numeric'},{'>=',0,'<=',wmax});
            case 'pngres'
                % set png resolution
                pngres = varargin{n+1};
            case 'kr'
                % set particles initial velocity
                Kr = varargin{n+1};
                validateattributes(Kr,{'numeric'},{'real','increasing'});
            otherwise
                error('Unknown property name %s',varargin{n})
        end
    end
end

% Get sampling data
[ExpData,NUSData] = Sampling();

% Copy experimental data
sample = ExpData{1};
SF = ExpData{2};
sz1 = ExpData{3}(1);
sz2 = ExpData{3}(2);
```

```
zff1 = ExpData{4}(1);
zff2 = ExpData{4}(2);
SF1 = ExpData{5}(1);
SF2 = ExpData{5}(2);
G = ExpData{6};
R = ExpData{7};
cenpts = ExpData{8};
winloc = ExpData{9};

% Copy NUS data
NUS_signal = NUSData{1};
R_scheme = NUSData{2};
G_scheme = NUSData{3};
spectra = NUSData{4};
amps = NUSData{5};

% Copy optimization parameters
N = OptParam{1};
maxite = OptParam{2};
maxrun = OptParam{3};
tol = OptParam{4};

% Validate experimental data
validateattributes(SF1,{'numeric'},{'>',0});
validateattributes(SF2,{'numeric'},{'>',0});
validateattributes(cenpts,{'numeric'},{'>',0,'integer'});
```

```
% Validate NUS data
validateattributes(NUS_signal,{'numeric'},{'finite','nonnan'});
validateattributes(R_scheme,{'numeric'},{'>=',0});
validateattributes(G_scheme,{'numeric'},{'>=',0});
validateattributes(spectra,{'numeric'},{'finite','nonnan'});
validateattributes(amps,{'numeric'},{'finite','nonnan'});

% Validate optimization data
validateattributes(N,{'numeric'},{'>=',1,'real','scalar'});
validateattributes(maxite,{'numeric'},{'>=',1,'real','scalar'});
validateattributes(maxrun,{'numeric'},{'>=',1,'real','scalar'});
validateattributes(tol,{'numeric'},{'real','>=',0,'<=',1});
% MAIN CALCULATIONS --------------------------------------------------%

% Calculate Kd boundaries
if isempty(Kr)
    fun = @(x) KLB(G,R,x);
    Kr(1) = fzero(fun,0.1);
    Kr(2) = min(Kr(1)+3,-1);
    fprintf('\nRange to search for Kd automatically set to ')
    fprintf('[10^%.2f, 10^%.2f]\n',Kr(1),Kr(2));
end

% Calibrate CIS boundaries
HmaxCIS = max(max(abs(amps(:,3:4))));
NmaxCIS = max(max(abs(amps(:,1:2))));
amps(:,3:4) = amps(:,3:4)*(0.5*SF/HmaxCIS);
```

```
amps(:,1:2) = amps(:,1:2)*(3*(SF/9.865)/NmaxCIS);

% Get maximum values of mother peaks
mom_max = Reference(spectra(:,:,1),winloc);

% Define time vectors
t1 = (0:sz1*zff1-1)./SF1;
t2 = (0:sz2*zff2-1)./SF2;

% Pre-allocate output
num = size(cenpts,1);
est = zeros(num,4);

% Correct peaks one by one
for pn = 1:num
    tic
    est(pn,:) = Correction(amps(pn,:),winloc(pn,:),mom_max(pn),tol);
    toc
end
% POST-PROCESSING ----------------------------------------------------%

% Remove outliers
Kavg = median(est(:,3));
Kstd = mad(est(:,3));
good = zeros(num,1);
for k = 1:num
    if abs(est(k,3)-Kavg)<2*Kstd
```

```
        good(k,1) = k;
        est(k,5) = 1;
    else
        est(k,5) = 0;
    end
end
good = nonzeros(good);
num = numel(good);
% SAVE RESULTS -------------------------------------------------------%

if nargout > 0
    res.sample = sample;
    res.estimations = est;
    res.signal = NUS_signal;
    res.Rscheme = R_scheme;
    res.Gscheme = G_scheme;
    res.spectra = spectra;
    res.CISamplitudes = amps;
    res.Nparticles = N;
    res.maxite = maxite;
    res.maxrun = maxrun;
    res.tolerance = tol;
    res.Krange = Kr;
    res.v0 = v0;
    res.c1 = c1;
    res.c2 = c2;
    res.wmax = wmax;
```

```
    res.wmin = wmin;
end
% PLOTTING ------------------------------------------------------------%

params = dlmread('classical_fit.txt');

Kfit = params(1,5);
err = params(1,6);
file = fopen('labels.txt');
L = textscan(file,'%s');
L = L{1};
fclose(file);

c1names = {'MarkerFaceColor'};
c1vals = {[0.3467 0.5360 0.6907]};
c2names = {'MarkerFaceColor'};
c2vals = {[0.9153 0.2816 0.2878]};
c3names = {'MarkerFaceColor','LineWidth'};
c3vals = {[0.3467 0.5360 0.6907],1.5};

figure()
subplot(2,2,1)
scatter(1:num,params(good,1),'o',c1names,c1vals),hold on
scatter(1:num,est(good,1),'s',c2names,c2vals),title('CIS1')
xlim([0.75 num+0.25])
ylim([min(amps(:,1)) max(amps(:,2))])
ylabel('\delta_{1} [Hz]')
```

```
set(gca,'XTick',1:num,'xticklabel',L(good),'XTickLabelRotation',45)
box on

subplot(2,2,3)
scatter(1:num,params(good,2),'o',c1names,c1vals),hold on
scatter(1:num,est(good,2),'s',c2names,c2vals),title('CIS2')
xlim([0.75 num+0.25])
ylim([min(amps(:,3)) max(amps(:,4))])
ylabel('\delta_{2} [Hz]')
set(gca,'XTick',1:num,'xticklabel',L(good),'XTickLabelRotation',45)
box on

subplot(2,2,[2 4])
errorbar(1:num,params(good,3),params(good,4),'o',c3names,c3vals),hold on
scatter(1:num,est(good,3),'s',c2names,c2vals),title('K_{d} values')
h = fill([0.75,num+0.25,num+0.25,0.75],...
    [Kfit-2*err,Kfit-2*err,Kfit+2*err,Kfit+2*err],'g',...
    'FaceAlpha',0.15,'EdgeColor','none');
[~,lg] = legend('Classical','NOSCO','95% CI classical');
PatchInLegend = findobj(lg, 'type', 'patch');
set(PatchInLegend(1), 'FaceAlpha', 0.15);
h.Annotation.LegendInformation.IconDisplayStyle = 'off';
line([0.75,num+0.25],[Kfit,Kfit],...
    'Color',c1vals{1},'LineWidth',2)
line([0.75,num+0.25],[mean(est(good,3)),mean(est(good,3))],...
    'Color',c2vals{1},'LineWidth',2)
xlim([0.75 num+0.25])
```

```
ylim([10^Kr(1) 10^Kr(2)])
ylabel('K_{d} [M]')
set(gca,'yscale','log','XTick',1:num,'xticklabel',L(good),...
    'XTickLabelRotation',45)
KstrC = sprintf('%.1e',Kfit);
EstrC = sprintf('%.1e',2*err);
KstrN = sprintf('%.1e',mean(est(good,3)));
EstrN = sprintf('%.1e',2*std(est(good,3)));
strC = ['Classical K_{d} = ',KstrC,'\pm',EstrC];
strN = ['NOSCO K_{d} = ',KstrN,'\pm',EstrN];
str = [strC char(10) strN];
dim = [0.59 0.15 0.1 0.1];
annotation('textbox',dim,'String',str,'FitBoxToText','on','FontSize',8);
box on

% Save output figure with auto-renaming
fname = sprintf('%s_figure',sample);
file = dir(strcat(fname,'*'));
count = size({file.name},2);
fname = strcat(fname,'_(',num2str(count+1),').png');
print(gcf,fname,'-dpng',pngres);

% Save output structure 'res' with auto-renaming
str = [sample,'_res*'];
file = dir(str);
count = size({file.name},2);
save([sample,'_res_(',num2str(count+1),').mat'],'res');
```

```
% FUNCTIONS ----------------------------------------------------------%

% Function to find Kd lower boundary
function val = KLB(G,R,x)
    P = G.*(1+R)+(10^x);
    Chi = (P-sqrt(P.^2-R.*(2*G).^2))./(2*G);
    val = 100*(Chi(end)-Chi(end-1))/Chi(end-1)-1;
end

% Read mother peaks maxs
function mom_max = Reference(momspec,winloc)

    mom_max = zeros(1,size(winloc,1));
    for i = 1:size(winloc,1)
        A = momspec(winloc(i,1):winloc(i,2),winloc(i,3):winloc(i,4));
        mom_max(i) = max(A(:));
    end

end

% Main correction function
function res = Correction(amps,winloc,mom_max,tol)

        function peak_max = local(winloc,mom_max,params)

            P = G_scheme.*(1+R_scheme)+params(3);
            Chi = (P-sqrt(P.^2-R_scheme.*(2*G_scheme).^2))./(2*G_scheme);
```

```
            arg_t1 = repmat(Chi.*t1',[1 2*sz2*zff2]);
            arg_t2 = repmat(Chi*t2,[2 1]);

            % This is the main block were the correction is applied (in
            % 2 steps to deal with the hypercomplex arrays)
            corr1 = NUS_signal.*exp(-1i*2*pi*(arg_t1*params(1)));
            data = [real(corr1);imag(corr1)];
            fidmocplx0 = data(:,1:2:end)+1i*data(:,2:2:end);
            corr2 = fidmocplx0.*exp(-1i*2*pi*arg_t2*params(2));
            fidmo = real(fftshift(fft(corr2,sz2*zff2,2),2));
            fidmocplx = fidmo(1:end/2,:)+1i*fidmo(end/2+1:end,:);
            spectrum = real(fftshift(fft(fidmocplx,sz1*zff1,1),1));
            peak = spectrum(winloc(1):winloc(2),winloc(3):winloc(4));
            peak_max = abs(mom_max - max(peak(:)));
        end

    % PSO
    func = @(params) local(winloc,mom_max,params);
    lb = [amps(1),amps(3),10^Kr(1)];    % Lower bounds
    ub = [amps(2),amps(4),10^Kr(2)];    % Upper bounds
    tol = tol*mom_max;                  % Tolerance

    [res,M] = pso(func,lb,ub,tol);      % Call PSO method
    res(1,4) = mom_max-M;
end

% PSO method
```

```
function [rfgbest,fffmin] = pso(fun,LB,UB,tol)

    if (tol == 0)
        fprintf('\nNo tolerance provided!')
    end

    m = numel(LB);
    fff = zeros(1,maxrun);
    rgbest = zeros(maxrun,m);

    % run loop --------------------------------------------------start
    for run = 1:maxrun
        % pso initialization
        for i = 1:N
            parfor j = 1:m-1
%                 rng('shuffle');
                x0(i,j) = LB(j)+rand()*(UB(j)-LB(j));
            end
        end
        % logarithmic distribution of initial K values
        Kinit = logspace(log10(LB(m)),log10(UB(m)),N);
        x0(:,m) = Kinit(randperm(N));

        x = x0;     % initial population, n x m
        v = v0*x0;  % initial velocity

        parfor i = 1:N
```

```
    f0(i) = fun(x0(i,:));
end
[fmin0, index0] = min(f0);
pbest = x0;             % initial pbest
gbest = x0(index0,:);   % initial gbest
ite = 0;
fprintf('\npeak %d, run %d/%d, iteration %d/%d - K = %.2e',...
    pn,run,maxrun,ite,maxite,gbest(1,3));

% iteration loop -----------------------------------------start
ite = 1;
while ite<=maxite && fmin0>tol
    fprintf('\npeak %d, run %d/%d, iteration %d/%d - ',...
        pn,run,maxrun,ite,maxite);

    w = wmax-(wmax-wmin)*ite/maxite; % update inertial weight

    % pso velocity updates
    parfor i = 1:N
        for j = 1:m
%                 rng('shuffle');
            v(i,j) = w*v(i,j)+c1*rand()*(pbest(i,j)-x(i,j))+...
                     c2*rand()*(gbest(1,j)-x(i,j));
            x(i,j) = x(i,j)+v(i,j);
        end
    end
```

```
% handling boundary violations
parfor i = 1:N
    for j = 1:m
        if x(i,j)<LB(j)
            x(i,j) = LB(j);
        elseif x(i,j)>UB(j)
            x(i,j) = UB(j);
        end
    end
end

% evaluating fitness
parfor i = 1:N
    f(i) = fun(x(i,:));
end

% updating pbest and fitness
parfor i = 1:N
    if f(i)<f0(i)
        pbest(i,:) = x(i,:);
        f0(i) = f(i);
    end
end

[fmin,index] = min(f0); % finding out the best particle

% updating gbest and best fitness
```

```
            if fmin<fmin0
                gbest = pbest(index,:);
                fmin0 = fmin;
            end
            fprintf('K = %.2e',gbest(1,3));
            ite = ite+1;
        end
        % iteration loop--------------------------------------------end

        fff(run) = fmin0;
        rgbest(run,:) = gbest;
        if fmin0 <= tol
            fprintf('\nDesired tolerance reached!')
            break
        else
        end
        fprintf('\nBest of run %d: K = %.2e',run,gbest(3));

    end
    % run loop -----------------------------------------------------end
    [fffmin,indexff] = max(fff); % finding out the best run
    rfgbest = rgbest(indexff,:);
    fprintf('\n Global best for peak %d - K = %.2e\n',pn,rfgbest(3));
end

end
```

References

(1) Casares, S.; Ab, E.; Eshuis, H.; Lopez-Mayorga, O.; Van Nuland, N. A.; Conejero-Lara, F. The high-resolution NMR structure of the R21A Spc-SH3:P41 complex: Understanding the determinants of binding affinity by comparison with Abl-SH3. *BMC Structural Biology* **2007**, *7*, 1–19.

(2) Berman, H. M. et al. The protein data bank. *Acta Crystallographica Section D: Biological Crystallography* **2002**, *58*, 899–907.

(3) Gonzalez-Magaña, A.; de Opakua, A. I.; Romano-Moreno, M.; Murciano-Calles, J.; Merino, N.; Luque, I.; Rojas, A. L.; Onesti, S.; Blanco, F. J.; De Biasio, A. The p12 subunit of human polymerase δ uses an atypical PIP box for molecular recognition of proliferating cell nuclear antigen (PCNA). *Journal of Biological Chemistry* **2019**, *294*, 3947–3956.

(4) Orekhov, V. Y.; Jaravine, V.; Mayzel, M.; Kazimierczuk, K. MddNMR - Reconstruction of NMR spectra from NUS signal using MDD and CS. **2004-2020**,

(5) Gołowicz, D.; Kasprzak, P.; Orekhov, V.; Kazimierczuk, K. Fast time-resolved NMR with non-uniform sampling. *Progress in Nuclear Magnetic Resonance Spectroscopy* **2020**, *116*, 40–55.

(6) Williamson, M. P. Using chemical shift perturbation to characterise ligand binding. *Progress in Nuclear Magnetic Resonance Spectroscopy* **2013**, *73*, 1–16.

(7) González-Ruiz, D.; Gohlke, H. Steering protein-ligand docking with quantitative NMR chemical shift perturbations. *Journal of Chemical Information and Modeling* **2009**, *49*, 2260–2271.

(8) Kennedy, J.; Eberhart, R. Particle swarm optimization. Proceedings of ICNN'95 - International Conference on Neural Networks. 1995; pp 1942–1948 vol.4.

(9) Kareem, L. A. Matlab Implementation of Particle Swarm Optimization. 2016.

(10) Nyquist, H. Certain topics in telegraph transmission theory. *Transactions of the American Institute of Electrical Engineers* **1928**, *47*, 617–644.

(11) Szántay, C. NMR and the uncertainty principle: How to and how not to interpret homogeneous line broadening and pulse nonselectivity. IV. Uncertainty. *Concepts in Magnetic Resonance Part A* **2008**, *32A*, 373–404.

(12) Tokunaga, Y.; Nagata, T.; Suetomi, T.; Oshiro, S.; Kondo, K.; Katahira, M.; Watanabe, T. NMR Analysis on Molecular Interaction of Lignin with Amino Acid Residues of Carbohydrate-Binding Module from Trichoderma reesei Cel7A. *Scientific Reports* **2019**, *9*.

(13) Mohanty, B.; Williams, M. L.; Doak, B. C.; Vazirani, M.; Ilyichova, O.; Wang, G.; Bermel, W.; Simpson, J. S.; Chalmers, D. K.; King, G. F.; Mobli, M.; Scanlon, M. J. Determination of ligand binding modes in weak protein–ligand complexes using sparse NMR data. *Journal of Biomolecular NMR* **2016**, *66*, 195–208.

(14) Gołowicz, D.; Kasprzak, P.; Kazimierczuk, K. Enhancing Compression Level for More Efficient Compressed Sensing and Other Lessons from NMR Spectroscopy. *Sensors* **2020**, *20*.

(15) Shchukina, A.; Urbańczyk, M.; Kasprzak, P.; Kazimierczuk, K. Alternative data processing techniques for serial NMR experiments. *Concepts in Magnetic Resonance Part A: Bridging Education and Research* **2017**, *46A*, e21429.

(16) Kazimierczuk, K.; Orekhov, V. Y. Accelerated NMR spectroscopy by using compressed sensing. *Angewandte Chemie - International Edition* **2011**, *50*, 5556–5559.

(17) Holland, D. J.; Bostock, M. J.; Gladden, L. F.; Nietlispach, D. Fast multidimensional

NMR spectroscopy using compressed sensing. *Angew. Chem. Int. Ed. Engl.* **2011**, *50*, 6548–6551.

(18) Hyberts, S. G.; Arthanari, H.; Wagner, G. Applications of non-uniform sampling and processing. *Topics in Current Chemistry* **2012**, *316*, 125–148.

(19) Delaglio, F.; Grzesiek, S.; Vuister, G. W.; Zhu, G.; Pfeifer, J.; Bax, A. NMRPipe: A multidimensional spectral processing system based on UNIX pipes. *Journal of Biomolecular NMR* **1995**, *6*, 277–293.
